# A Phase II, Randomized, Double-Blind, Placebo Controlled, Dose-Response Trial of the Melatonin Effect on the Pain Threshold of Healthy Subjects

**DOI:** 10.1371/journal.pone.0074107

**Published:** 2013-10-02

**Authors:** Luciana Cadore Stefani, Suzana Muller, Iraci L. S. Torres, Bruna Razzolini, Joanna R. Rozisky, Felipe Fregni, Regina Markus, Wolnei Caumo

**Affiliations:** 1 Post-Graduate Program in Medical Sciences, School of Medicine, Universidade Federal do Rio Grande do Sul, Porto Alegre, Rio Grande do Sul, Brazil; 2 Anesthetist, Pain and Palliative Care Service at Hospital de Clínicas de Porto Alegre, Laboratory of Pain and Neuromodulation, Universidade Federal do Rio Grande do Sul, Porto Alegre, Rio Grande do Sul, Brazil; 3 Associate Professor, Pharmacology Department, Instituto de CiênciasBásicas da Saúde, Universidade Federal do Rio Grande do Sul, Porto Alegre, Rio Grande do Sul, Brazil; 4 Laboratory of Chronopharmacology, Department of Physiology, Institute of Bioscience, University of São Paulo, São Paulo, Brazil; 5 Associate Professor of Physical Medicine and Rehabilitation, Associate Professor of Neurology Harvard Medical School. Berenson-Allen Center for Noninvasive Brain Stimulation, Department of Neurology, Beth Israel Deaconess Medical Center, Harvard Medical School, Boston, Massachusetts, United States of America; California Pacific Medicial Center Research Institute, United States of America

## Abstract

**Background:**

Previous studies have suggested that melatonin may produce antinociception through peripheral and central mechanisms. Based on the preliminary encouraging results of studies of the effects of melatonin on pain modulation, the important question has been raised of whether there is a dose relationship in humans of melatonin on pain modulation.

**Objective:**

The objective was to evaluate the analgesic dose response of the effects of melatonin on pressure and heat pain threshold and tolerance and the sedative effects.

**Methods:**

Sixty-one healthy subjects aged 19 to 47 y were randomized into one of four groups: placebo, 0.05 mg/kg sublingual melatonin, 0.15 mg/kg sublingual melatonin or 0.25 mg/kg sublingual melatonin. We determine the pressure pain threshold (PPT) and the pressure pain tolerance (PPTo). Quantitative sensory testing (QST) was used to measure the heat pain threshold (HPT) and the heat pain tolerance (HPTo). Sedation was assessed with a visual analogue scale and bispectral analysis.

**Results:**

Serum plasma melatonin levels were directly proportional to the melatonin doses given to each subject. We observed a significant effect associated with dose group. Post hoc analysis indicated significant differences between the placebo vs. the intermediate (0.15 mg/kg) and the highest (0.25 mg/kg) melatonin doses for all pain threshold and sedation level tests. A linear regression model indicated a significant association between the serum melatonin concentrations and changes in pain threshold and pain tolerance (*R^2^* = 0.492 for HPT, *R^2^* = 0.538 for PPT, *R^2^* = 0.558 for HPTo and *R^2^* = 0.584 for PPTo).

**Conclusions:**

The present data indicate that sublingual melatonin exerts well-defined dose-dependent antinociceptive activity. There is a correlation between the plasma melatonin drug concentration and acute changes in the pain threshold. These results provide additional support for the investigation of melatonin as an analgesic agent.

**Brazilian Clinical Trials Registry (ReBec)**: (*U1111-1123-5109*).

**IRB**: Research Ethics Committee at the Hospital de Clínicas de Porto Alegre.

## Introduction

Melatonin is the major hormone synthesized by the pineal gland and is mainly involved in the control of circadian rhythm [1]. However, melatonin is also involved in the regulation of other systems such as the pain system. In fact, the antinociceptive effect of melatonin has been demonstrated in animal models of acute pain [2], [3], inflammatory pain [4] and neuropathic pain [5] Preliminary studies in humans have shown melatonin effects on some pain syndromes, especially fibromyalgia [6] and acute postoperative pain [7], [8], [9]. Despite these initial positive results, the dose-response effect of melatonin on pain has not been explored.

Melatonin interacts with two receptors (MT1 and MT2) at different sites in the brain [10]. However, its antinociceptive effects are not fully understood. Previous studies have suggested that this hormone could have an effect on the spinal cord and thus alter nociceptive transmission at this level [11]. Melatonin also has modulatory functions on opioid and gamma-aminobutyric acid (GABA) systems [12], [13], [14]. Furthermore, melatonin may have a peripheral effect as shown by inhibitory activity on the release of pro-inflammatory cytokines at peripheral sites [15]. Therefore, melatonin may mediate antinociception through peripheral and central mechanisms. Based on the preliminary encouraging results of studies of the effects of melatonin on pain modulation, the important question of whether there is a dose relationship in humans of melatonin on pain modulation has been raised. To fill this gap in knowledge, we decided to test the hypothesis that melatonin would have a dose-response antinociceptive effect. To measure pain in this study, we chose to measure pain threshold and tolerance. Although the pain threshold is fairly constant, the pain tolerance level, which is defined as the amount of pain a subject is prepared to put up with, varies enormously. Interestingly, patients do not usually seek medical advice until they are beyond their pain tolerance. For a given noxious stimulus, the intensity with which pain is felt varies from person to person, and a distinction has to be made between an individual's pain threshold and pain tolerance [16]. This was defined in experimental pain models in healthy human volunteers by measuring analgesic drug effects using a noninvasive, non-noxious, standardized and repeatedly applicable stimulus[17].

Thus, we investigated the melatonin dose-response effect on pain threshold after considering the inter-individual and intra-individual variability. We tested the pressure pain tolerance, the heat pain tolerance and the sedative effect. The effect of melatonin on pain measurement was adjusted by the sedation level.

## Materials and Methods

The protocol for this trial and supporting CONSORT checklist are available as supporting information; see Checklist S1 and Protocol S1.

### Subject and study design

After obtaining approval from the Research Ethics Committee of the Hospital de Clínicas de Porto Alegre, 61 white healthy volunteers with a mean age of 26.8 y (the range was 19–47 y) were enrolled into the randomized, parallel, double-blind, placebo-controlled study. The study was conducted in accordance with the Declaration of Helsinki on biomedical research involving human subjects, and written informed consent was obtained from the participants.

The volunteers were recruited from the general population by postings in universities, on the Internet and in public places in the Porto Alegre area. Subjects were considered eligible to participate if they were aged between 19 and 49 y. Interested individuals were screened for eligibility by phone. They answered a structured questionnaire assessing the following variables: current acute or chronic pain conditions, use of analgesics in the past week, rheumatologic disease, clinically significant or unstable medical or psychiatric disorder, history of alcohol or substance abuse in the past 6 months, neuropsychiatric comorbidity, and use of central nervous system-affecting medications. Patients responding affirmatively to questions about any of these conditions were excluded from the study. In Brazil, economic incentives for research participation are not allowed.

### Sample size justification

The number of subjects in each study group was determined based on our previous study [18]. An a priori estimate indicated that a total sample size of 60 divided in four balanced treatment groups (n = 15) was required to detect an increase of 1.31 kg/cm^2^/second in pain pressure threshold [mean standard deviation (SD) 0.9 kg/cm^2^/second] in the melatonin group and a difference of 2.5 [mean SD 3°C (Celsius)] in heat pain threshold, with a power of 0.8 and an α level of 0.05. The sample size estimated a priory was defined by pain threshold (pressure and heat) considering that across healthy individuals it is expected a higher variability in pain threshold compared with the pain tolerance [19]. However, the power of analysis for the pain threshold (heat and pressure), as well as for pain tolerance (pressure and heat) it was higher than 80% with a 2-tailed with an α-error of 0.01.

### Study plan

The data were collected at the Clinical Research Center of the Hospital de Clínicas de Porto Alegre. The volunteers were asked to abstain from alcohol and excessive coffee consumption (defined as at least 5 cups of coffee day, a dose that corresponds to 400 mg of caffeine) for 24 h before testing and from drinking and eating for 6 h before testing. Study sessions were performed in a quiet, non-stressful environment at the same air-conditioned location, and the sessions always started at the same time in the afternoon. The volunteers rested comfortably in a semi-recumbent position during the experiments and were monitored with non-invasive blood pressure, pulse oximetry and a BIS (bispectral index) Quatro Sensor (Aspect Medical Systems model A-2000; Aspect Medical Systems, Newton, MA), which was applied on the forehead and connected to the BIS monitor. After a trial run to familiarize the volunteers with the procedures, two test series were performed: at baseline and 30 minutes after the intervention. The subjects were always assessed by the same researcher (L. Stefani), an anesthesiologist with extensive experience in clinical and experimental pain assessment, who systematically read the instructions and explained the standardized experimental procedure using a modified previously published protocol [19] for quantitative sensory testing (QST) assessment. They received instructions to pushing the rescue button only when they actually get painful. After the test series, a venous blood sample was taken for analysis of the plasma melatonin concentration. The blood samples were immediately stored at 4°C and centrifuged at 3500 rpm for 10 min after the last test series. The plasma was frozen at −80°C for later analysis.

### Interventions

The intervention involved one of three doses of sublingual melatonin (Sigma Chemical, Germany, batch-by-batch certificates of analysis for authenticating the purity of each batch provided): 0.05 mg/kg, 0.15 mg/kg, 0.25 mg/kg (maximum dose 20 mg), or placebo. The preparation of the melatonin solution was performed using propylene glycol (PG) (40%) plus cyclodextrins (30%) [20]. The dose-adjustment was performed by changing the concentration of melatonin in the vehicle. This solution was combined with 0.5 ml of 10% glucose solution. The placebo was an equivalent volume of 10% glucose solution. The nurse prepared the study drugs using the melatonin solution (20 mg/ml) in needleless syringes marked only with coded labels to maintain the double-blinded nature of the study. Patients received oral and written instructions that they could not swallow the liquid nor talk while the liquid was in their mouth.

### Randomization and blinding

We used a fixed block size of 12 to ensure that equal numbers of participants were randomized into the four groups. A computer random number generator stratified by gender assign the patients to one of three melatonin doses or the placebo. Before the recruitment phase, opaque envelopes containing the allocated treatment were sealed and numbered sequentially. The envelopes were only allowed to be opened after the subject signed the consent form; the nurse who administered the medications opened the envelopes – this nurse was not involved in other components of the trial; in fact, investigators who conducted the experiment and evaluators did not participate in enrollment decisions. Two investigators who were not involved in subject evaluation performed randomization. Other individuals who were involved in patient care were unaware of the treatment group to which the patients belonged.

### Outcomes

The main primary outcome was the heat pain threshold and the pressure pain threshold. Pain tolerance was considered as the main secondary outcome and the sedation level as another secondary outcome. The pain threshold, pain tolerance and sedation level as reported using a VAS were measured at baseline and 30 min after administration of the medication. The sedation level was also measured continuously using the BIS for 30 min.

### Experimental pain tests

#### Heat pain thresholds and tolerance

Quantitative sensory testing (QST) was used to assess heat pain thresholds using the method of limits with a computer Peltier-based device thermode (30×30 mm) [21]. The thermode was attached to the skin on the ventral aspect of the mid-forearm. The baseline temperature was set at 32°C and was increased at a rate of 1°C/s to a maximum of 52°C. This slow rise time was selected as a test of pain, which is primarily evoked by stimulation of C-nociceptive afferents as previously demonstrated [22]. Each participant was asked to press a button as quickly as possible at the moment the stimulation became painful. Three assessments were taken with an interstimulus interval of 40 s [23], and the thresholds were calculated by taking the average temperature of the three assessments. The position of the thermode was slightly altered between trials (although it remained on the left ventral forearm) to avoid either sensitization or the response suppression of cutaneous heat nociceptors. The same equipment was used to determine the maximally tolerated temperature on the ventral aspect of the mid forearm. Starting at a baseline temperature of 32°C, the thermode was heated at a rate of 1.0°C/s. The volunteer pressed a button when he/she did not want the temperature to be increased any further (pain tolerance is the maximal temperature that a person is able to tolerate; the cutoff limit was 52°C), which caused the thermode to cool to the baseline temperature. When the heat was raised up to a maximum of 52°C and the subject did not feel pain at that temperature, the real pain threshold was considered unknown.

#### Pressure pain test

A Fisher's pressure algometer (Pain Diagnostics and Thermography, Great Neck, NY 11023) [24] was used to determine pain pressure detection and pain pressure tolerance. The pressure was gradually increased at a rate of 1 kg/cm^2^/second. A probe with a surface area of 1 cm^2^ was applied perpendicular to the tibial surface. Prior to the test trial, the volunteer learned to differentiate the perception of pressure from the perception of the onset of pain. The subject was then instructed to verbally report the perception of pain onset. Subjects were asked to say ‘stop’ immediately after a discernible sensation of pain (distinct from pressure or discomfort) was felt. At this point, the experimenter immediately retracted the algometer [24]. The average value of three successive readings taken at intervals of 3–5 min was recorded as the pain pressure threshold [24]. We also measured the pain pressure tolerance, which was defined as the maximally tolerated pressure applied to the tibial surface. Subjects were asked to say ‘stop’ when they did not want the pressure to be increased any further (pain pressure tolerance is the maximum level of pressure that a person is able to tolerate; the cutoff limit was 10 kg/cm^2^).

The clinical assessment of sedation was determined by simultaneous recording using a visual analogue scale (VAS) ranging from zero (awake) and 100 mm (complete sleepiness). The BIS was obtained with BIS (model A-2000; Aspect Medical Systems, Newton, MA). After electrode placement above the bridge of the nose, over the temple area, and between the corner of the eye and the hairline, the monitor initiated automatic impedance testing to ensure acceptable signal reception. The electrodes were repositioned or replaced if impedances increased enough to impair the EEG evaluation. A reading of zero indicated no brain activity, and a reading of 100 indicated a fully awake state. The BIS score correlates quantitatively with the alertness of sedated patients without being confounded by evaluator or patient bias [25]. Data from the BIS monitor were recorded for the study group for 30 min after melatonin administration.

### Assessment of demographic characteristics, depressive symptoms and anxiety

All of the tests used in the present study were validated for the Brazilian population and performed in the presence of a previously trained evaluator.

- Demographic data were assessed using a standardized questionnaire.
- Depressive symptoms were assessed by the Beck Depression Inventory (BDI). The final scores ranged from 0 to 63 [26].
- Anxiety was measured with the refined version of the Rasch analysis of the State-Trait Anxiety Inventory (STAI), which was adapted to Brazilian Portuguese [27]. State anxiety (a situation-driven transient anxiety) and trait anxiety (stable personality disposition reflecting general level of fearfulness) were evaluated. Each state item was given a weighted score of 1 to 4. The total number of items was 13, and the possible scores range was 13 to 52. Each trait item was given a weighted score of 1 to 3. The total number of items was 12, and total possible score ranged was from 12 to 36. Higher scores denoted higher levels of anxiety.

### Melatonin determination

The blood samples were centrifuged in plastic tubes for 10 min at 3500× g at 4°C, and the serum was stored at −80°C for the hormone assays. To assess bioavailability, serum melatonin was determined by ELISA using commercial kits from MP Biomedical Inc. (Irvine, California, USA) that employed the basic principles of competitive immunoassays [28]. The detection limit of the ELISA assay was 0.3 ng/mL (300 pg/ml).

### Statistical analyses

The differences between the groups on baseline were examined by analysis of variance (ANOVA) for parametric variables, and categorical variables were examined by chi-square or Fisher's exact tests given that our main independent outcome (intervention) was also categorical. Linear regression and slope analysis were performed to obtain the serum melatonin concentration and pain threshold relationship. A Spearman correlation coefficient (*r_s_*) was used to assess the correlation between VAS and BIS sedation scores with pain threshold and pain tolerance.

The results were evaluated using the absolute mean variation on heat pain thresholds and pressure pain threshold, heat and pain pressure tolerance and on scores of delta values (post-treatment minus pre-treatment). Given the several outcomes of pain threshold levels did not present normal distribution we analyzed data using Kruskal-Wallis test with Dunn's Multiple Comparison Test to identify changes between groups. For the BIS data (obtained each minute during a 30-min period), we conducted a group analysis with a mixed ANOVA model in which the independent variables were time, treatment (placebo vs. melatonin 0.05, 0.15 and 0.25 mg), the interaction term time vs. the treatment group and subject ID. We performed post hoc analysis using paired t tests to assess the effects of each treatment group. To ensure normally distributed data, we performed a log transformation for pain threshold (heat and pressure), pain tolerance (heat and pressure), as well the serum plasma melatonin level to assess the relationship between serum plasma melatonin levels and analgesic effects. Within-group the standardized mean difference (SMD) was computed in terms of the ratio between the mean change and the baseline standard deviation. The SMD was interpreted as follows: small, 0.20; moderate, 0.50–0.60 and large, 0.80[29]. All of the analyses were performed in assuming intention-to-treat, hence including all of the randomized subjects for whom there were observations in the study outcomes.

Formal testing of observed and unobserved bias (on the assumption that blinding may not have been completely effective) using the Berger-Exner test [30] were also performed. The analyses were performed with GraphPad Prism version 4.00 for Windows (GraphPad Software, USA) and SPSS version 18.0 (SPSS, Chicago, IL).

## Results

**Figure 1:**
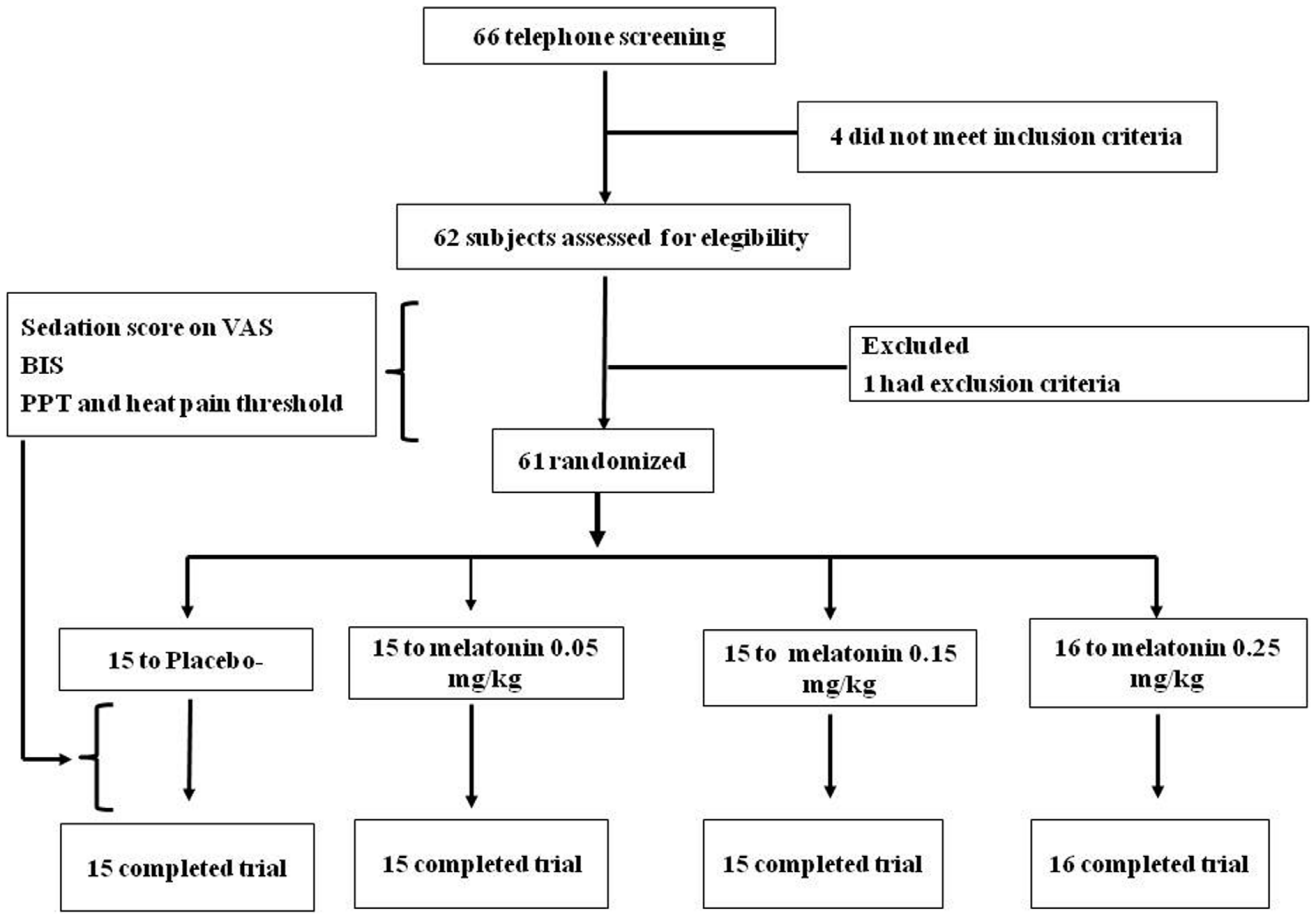
Flow and number of patients in each phase of the study.

Sixty-one subjects were randomized into one of four groups (**Figure 1**). The subject characteristics are summarized in **Table 1**. The demographic characteristics are shown for each group of subjects assigned to receive one of three doses of melatonin or placebo. The ages of the subjects and the gender distribution did not differ between the four groups. The Berger-Exner tests supports the lack of evidence for observed and unobserved bias in treatment group assignment. Controlling for treatment arm, the P-values for the partial correlation between heat pain threshold and pain pressure threshold (the main outcomes) and the dose-group in the block at the time of random assignment to therapy was >0.4 in each of the four groups.

**Table 1:** Demographic data and psychological profiles (n = 61).

Variable	Placebo	Melatonin	Melatonin	Melatonin	*F*	*P*
		0.05 mg/kg	0.15 mg/kg	0.25 mg/kg		
	(n = 15)	(n = 15)	(n = 15)	(n = 16)		
Male/Female €	7/8	7/8	7/8	8/8	__	
Age (years) †	25.13 (5.50)	25.2 (5.07)	25 (6.44)	23.68 (2.72)	4.7	0.72
Body index (kg/m^2^) †	22.50 (4.03)	24.16 (6.47)	22.76 (3.29)	22.75 (2.71)	0.92	0.43
Beck Depressive Inventory						
symptoms †	3.2 (2.54)	4.8 (5.7)	4.0 (2.73)	3.37 (2.87)	0.57	0.63
Trait Anxiety †	21.13 (3.77)	22.26 (5.6)	19.85 (3.15)	18.54 (4.59)	0.09	0.96
State Anxiety †	19.0 (3.0)	18.93 (5.4)	19.21(2.63)	20.25 (4.2)	0.92	0.98

† Compared by ANOVA; € compared using Chi-Square.

### Between-group changes in pain threshold and sedation

This study no find significant correlation between the pain threshold and pain tolerance with sedation score and the BIS value, as presented in **Table 2**.

**Table 2:** Correlation between sedation level with pain threshold and pain tolerance (n = 61).

	BIS index	HPT(°C)	HPT (°C)	PPT	PPTo
				(Kg/cm^2^/second)	(Kg/cm^2^/second)
**VAS sedation score**	−0.32	0.18	0.20	0.14	0.27
	*(P = 0.03)* *	*(P = 0.17)*	*(P = 0.15)*	*(P = 0.45)*	*(P = 0.15)*
**BIS index**	–	−0.07	−0.10	−0.21	−0.24
		*(P = 0.60)*	*(P = 0.42)*	*(P = 0.08)*	*(P = 0.05)*

*Spearman correlation coefficient (r_s_) is significant at the 0.05 level (2-tailed).

Mean of the BIS at 30 min after medication (BIS index); mean Delta sedation score on the VAS (VAS sedation score); heat pain threshold (HPT); score on heat pain tolerance (HPTo); pain pressure threshold (PPT); mean Delta pressure pain tolerance (PPTo).

The results of the effects of melatonin on pain threshold, pain tolerance, sedation scores as well in BIS are shown in **Table 3**. The post hoc analysis indicated significant differences between the placebo and the intermediate and highest melatonin doses in all of the tests.

**Table 3:** The mean delta score (SD) (post-treatment values minus pre-treatment values) of the pressure and heat pain threshold, the pressure pain tolerance (PPTo) and the heat pain tolerance (HPTo) tests or sedation score (n = 61).

Treatment	Mean (SD)	Mean (SD)	Median	*P*	SDM
		(Delta)	(Quartile 75;25)		
**Pain pressure threshold(kg/cm2/second)** †					
Placebo (n = 15)	6.71 (1.57) *vs.* 6.64 (1.50)	0.08 (0.03) ^a^	0.40 (0.23; 0.90)	0.001	0.05
Melatonin 0.05 mg/kg (n = 15)	6.79 (1.54) *vs.* 6.59 (1.54)	0.20 (0.35) ^a^	0.98 (0.27; 1.60)		0.13
Melatonin 0.15 mg/kg (n = 15)	6.48 (1.89) *vs.* 5.54 (1.58)	0.94 (0.79) ^b^	1.06 (0.65; 1.43)		0.50
Melatonin 0.25 mg/kg (n = 16)	7.37 (1.74) *vs.* 6.02 (1.64)	1.35 (1.26) ^b^	1.06 (0.66; 1.43)		0.78
**Heat pain threshold (°C)** †					
Placebo (n = 15)	43.23 (2.51) *vs.*42.90 (2.43)	0.33 (2.11) ^a^	0.67 (0.37; 1.10)	0.001	0.13
Melatonin 0.05 mg/kg (n = 15)	43.10 (2.42) *vs.* 42.56 (2.37)	0.54 (0.96) ^a^	2.20 (1.41; 2.96)		0.37
Melatonin 0.15 mg/kg (n = 15)	44.71 (2.91) *vs.* 42.75 (3.15)	1.96 (1.08) ^b^	2.38 (1.72; 3.65)		0.67
Melatonin 0.25 mg/kg (n = 16)	45.05 (3.05) *vs.* 42.56 (3.56)	2.49 (2.88) ^b^	2.38 (1.72; 3.65)		0.82
**Score on heat pain tolerance (HPTo) (°C)** †					
Placebo (n = 15)	43.23(2.16) *vs.* 42.50 (2.49)	0.32 (0. 8) ^a^	0.87 (0.73; 1.08)	0.001	0.15
Melatonin 0.05 mg/kg (n = 15)	43.10(2.22) *vs.* 42.28 (2.32)	0.82 (0. 84) ^a^	1.36 (1.03; 1.56)		0.40
Melatonin 0.15 mg/kg (n = 15)	44.91 (2.71) *vs.*42.68 (3.15)	2.23 (0.49)^a, b^	1.87 (1.53; 2.43)		0.83
Melatonin 0.25 mg/kg (n = 16)	45.05 (3.05) *vs.*41.74 (3.56)	3.31 (0.73) ^b^	1.98 (1.70; 2.31)		1.09
Mean delta pressure pain tolerance (kg/cm2/second)†					
Placebo (n = 15)	6.89(1.87) *vs*. 6.08 (1.50)	0.81 (0.57) ^a^	0.90 (0.63; 1.17)	0.001	0.43
Melatonin 0.05 mg/kg (n = 15)	7.38 (1.74) *vs*. 6.59 (1.54)	0.79 (0.74) ^a, b^	1.24 (0.90; 1.58)		0.45
Melatonin 0.15 mg/kg (n = 15)	8.29 (1.98) *vs*. 6.89 (1.58)	1.40 (0.82) ^a, b^	1.61 (1.50; 2.18)		0.70
Melatonin 0.25 mg/kg (n = 16)	8.43 (1.74) *vs*. 6.98 (1.64)	1.45 (0.78) ^b^	2.04 (1.76; 2.19)		0.83
**Mean delta sedation score on the VAS** †					
Placebo (n = 15)	2.19 (1.41) *vs*.1.21 (0.97)	0.98 (1.76) ^a^	.19 (0.07; 2.47)	0.16	0.70
Melatonin 0.05 mg/kg (n = 15)	3.48 (1.87) *vs*.1.65 (1.82)	1.83 (1.14) ^a^	1.57 (0.53; 2.54)		0.98
Melatonin 0.15 mg/kg (n = 15)	3.82 (1.94) *vs*. 1.45 (1.62)	2.37 (1.70) ^a^	2.53 (1.28; 3.48)		1.22
Melatonin 0.25 mg/kg (n = 16)	4.47 (1.51) *vs*. 1.78 (1.52)	2.69 (1.50) ^b^	2.53 (1.65; 4.28)		1.78
**Mean of the BIS during 60 min after medication** *$*					
Placebo (n = 15)	97.42 (1.84) *vs.* 97.63 (2.0)	−0.21 (1.70) ^a^	7.28	0.0001	0.11
Melatonin 0.05 mg/kg (n = 15)	97.21 (1.93) vs. 97.92 (2.43)	−0.71 (2.07) ^b^			0.37
Melatonin 0.15 mg/kg (n = 15)	96.17 (1.82) vs. 97.42 (2.21)	−.1.25 (1.82) ^c^			0.69
Melatonin 0.25 mg/kg (n = 16)	94.75 (2.94) vs. 97.14 (1.70)	−2.39 (1.70) ^c^			0.82

Visual analogue scale (VAS).

Different superscripts (a, b, and c) indicate significant differences among treatment groups according to the Bonferroni test.

† Kruskal-Wallis test with Dunn's Multiple Comparison Test to identify changes between groups.

$ Mixed ANOVA model.

Standardized mean difference (SMD) [(pre minus post)/baseline standard deviation]. The size effect was interpreted as follows: small, 0.20;, moderate, 0.50–0.60 and large, 0.80.

### Dose-concentration curve for the pain threshold

**Figure 2:**
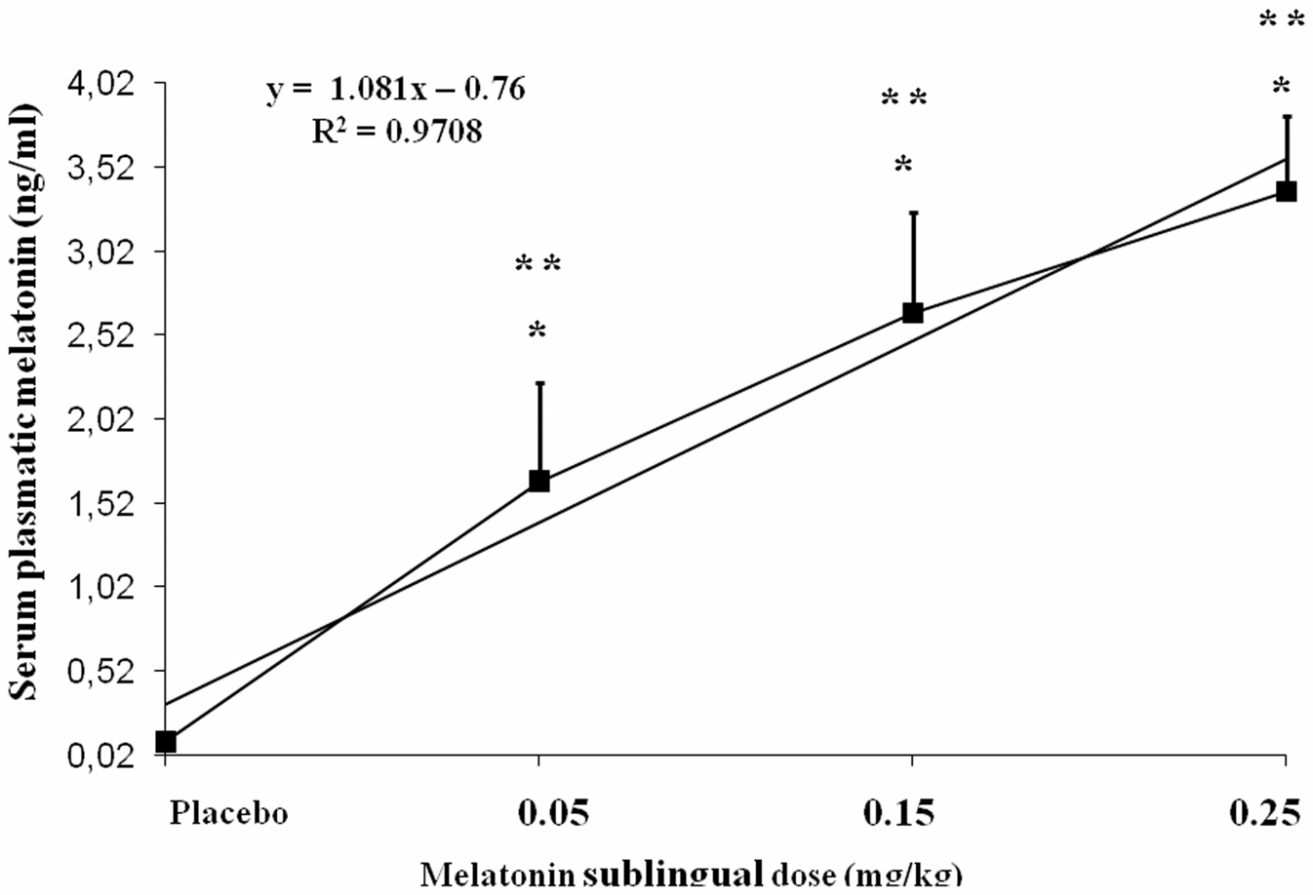
Dose-concentration curve comparing the mean concentration achieved at 30 min following sublingual doses of 0.05, 0.15 or .25 mg/kg (n = 61). Serum plasma melatonin level at 30(P<0.05) at the time points. (*) Differences between placebo and melatonin. (**) Differences between 0.05, 0.15, and 0.25 mg/kg doses. All comparisons were made using a regression analysis model, followed by Bonferroni test for post-hoc multiple comparisons. *F(3∶57)* = 127; (P<0.0001); *R^2^* = 0.86.

The dose-concentration response curve for melatonin was subproportional to the dose given to each patient. Variations in sublingual melatonin doses accounted for 97% of the variance in plasma melatonin concentrations (**Figure 2**). This figure presents the dose-concentration curve generated from three different doses of melatonin, which can be used to determine potency and maximal efficacy.

**Figure 3:**
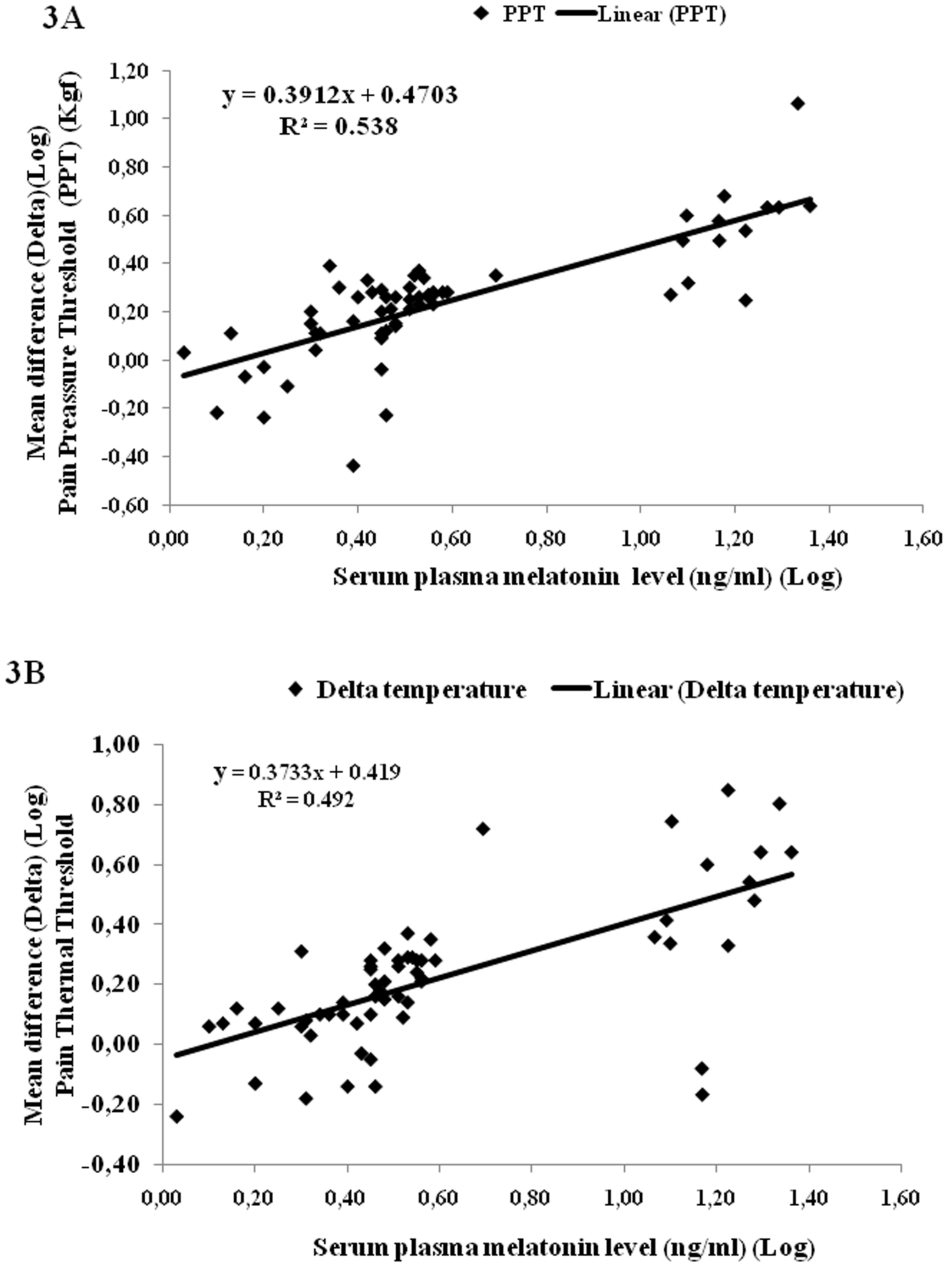
Effect of serum plasma melatonin of all of the volunteers on pressure pain threshold (A) and heat pain threshold (B) (n = 61).

**Figure 4:**
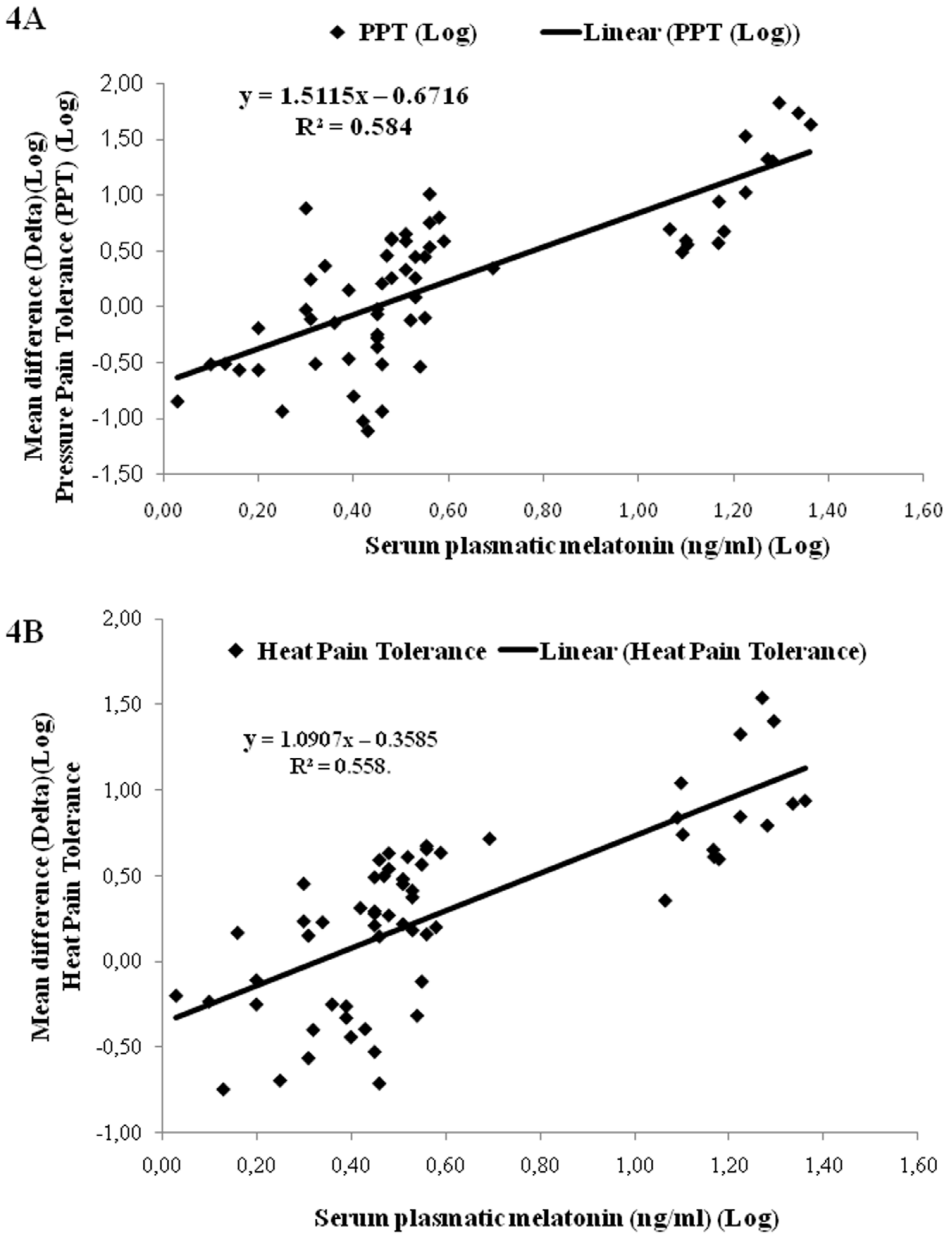
Effect of serum plasma melatonin of all of the volunteers on pressure pain tolerance (A) and heat pain tolerance (B) (n = 61).

Comparisons of the concentration indicate a linear relationship between serum plasma melatonin levels and analgesic effects (**Figures 3A,B**; 4 A,B). The correlations between the serum melatonin concentrations and the change in the outcome variables (pain threshold and pain tolerance) are derived from the linear regression model. The melatonin serum concentration accounted for 53.8% and 49.2% of the variance of pain pressure threshold and heat pain threshold, respectively (**Figures 3 A, B**).

The melatonin serum concentration accounted for 58.4% and 55.8% of the variance of pain pressure tolerance and heat pain tolerance, respectively (**Figures 4 A, B**). Eight of the subjects (13.3%) did not feel pain even after heating up to the maximum of 52°C, hence their real pain threshold remained unknown.

## Discussion

The present study presented dose-concentration curves for the effects of melatonin on heat and pressure pain tests. Serum melatonin concentrations are within the normal dynamic range for dose melatonin concentrations (**Figure 2**) with rapid elevation, which indicates that the rate and extent of absorption of melatonin (bioavailability) is linear with the dose after sublingual administration. Interestingly, sublingual melatonin induced a dose-dependent analgesic effect on pain threshold and pain tolerance. A single dose of at least 0.15 mg/kg produced a significant increase in both pain measurements (threshold and tolerance) for heat and pressure (**Table 3**). Higher doses produced size effect increases in both pain threshold and pain tolerance (**Table 3**). The subjects in the present study did not experience any side effects besides sedation, which suggests that the different doses that were used were adequate but not excessive. The subjects who received 0.15 mg/kg or 0.25 mg/kg of sublingual melatonin presented a statistically significant increase in sedation (based on the BIS data) compared with those who received placebo or the lowest melatonin dose (**Table 3**).

The data obtained in the present study are best described with a dose-concentration curve. Our findings distinguish between inter-individual and intra-individual variability of the drug effect, which may be important for the interpretation of the results. The comparisons of mean delta also allowed for determinations of the dose-response effect for each individual. This effect was revealed and maintained independently of the type of stimulus applied (pressure or thermal). The consistency of the findings of the present study supports the hypothesis of a melatonin dose-response effect. The experiment was conducted in a controlled setting, which excludes the influence of several confounding factors observed in clinical pain, such as baseline pain, psychological factors and the presence of other analgesics.

The present findings corroborate positive results reported for melatonin in previous clinical studies assessing acute pain and chronic pain [6], [8], [9], as well in animal models [31] of nociceptive pain, such as the tail-flick [32], hot-plate [14], [33], tail electric stimulation and hind paw pinch [34] tests. Although a dose-dependent antinociceptive effect has been shown in some previous preclinical studies [13], [32], even with small melatonin doses, we cannot perform direct comparisons of dose-response curves between animal and human models because several aspects related to the varying doses (20 to 200 mg/kg), route of administration (intraperitoneal or intracerebroventricular) and kinetic parameters are species specific.

In the present study, the effect on nociceptive pain may be observed through changes in the thermal and pressure pain threshold, as well in pain tolerance. When comparing the effect on SMD presented in Table 3, it is possible to see that a dose of 0.15 mg/kg increased PPTo 28.57% (0.5 vs. 0.7) compared to the PPT test effect. This increase was 19.28% (0.67 vs. 0.83) for HPTo compared to the effect on HPT, respectively. Although such increment was relatively small, it was equivalent to the analgesic effect observed in other trials comparing active treatment with placebo [35].

Interestingly, studies have suggested that melatonin is synthesized in a number of extrapineal sites, including the spinal cord, which indicates that this hormone could act as a paracrine signal in addition to its endocrine action [36]. Furthermore, melatonin receptors are abundant in the spinal cord [37]. Thus, melatonin could play a role in the modulation of nociceptive transmission at this level [11]. Melatonin also modulates opioid and gamma-aminobutyric acid (GABA) systems [12], [13], [14], but it is not possible to dissociate the effect of each individual neurobiological system in human experimental and clinical studies. Indeed, only the net effect can be assessed. Studies have also suggested that additional pathways play a role in the analgesic actions of melatonin, such as nuclear signaling pathways, receptor-independent radical scavenging, and inhibition of the release of proinflammatory cytokines at peripheral sites. In addition, the highly lipid-soluble nature of melatonin allows it to easily penetrate the blood-brain barrier. Therefore, melatonin may cause antinociception through both peripheral and central mechanisms, and the effect may be related to dose, as shown in present study (**Table 3**). In the present study, the BIS value decreased with increasing melatonin dose. Although there were significant differences among the sedation levels of the individual groups, we do not believe that the differences are clinically significant. The magnitude of the differences is small because only a mild level of sedation was observed, and all of the subjects were easy aroused by verbal stimuli throughout the study. This hypothesis is supported by our findings, as neither pain threshold nor pain tolerance scores were altered by sedation level (**Table 2**). Accordingly, to obtain non-invasive and objective information about the clinical sedative effect, we used a BIS. The BIS permitted us to identify if a drug induced sedation in a dose-dependent manner, without the interference of evaluator bias[38]. However, further studies should assess if the sedation level observed with these doses has an impact on function in daily life because sedation is a major problem with most currently available pain drugs (i.e., antidepressants, anticonvulsants, and opioids).

It is important to assess the strengths and limitations of the clinical trial we conducted. We conducted this trial according to the CONSORT guidelines and given that we used the Delphi List (a criteria list for quality assessment of RCTs), our trial can be considered to be of strong quality because all eight items in this scale can be positively scored in our RCT [39]. However, some methodological choices should be taken into account in the interpretation of these findings. ***i***) It is important to emphasize that the methodology used does not allow the determination of the duration of effect. ***ii***) It is also important to consider that because of inter-individual pharmacokinetics, we may have missed the peak effect in some subjects. ***iii***) The permuted blocks method of randomization was used, although it has been described that such method could allow for prediction of future allocations. Thus, formal testing for selection bias (on the assumption that blinding may not have been completely effective) using the Berger-Exner test was performed. This test found no evidence of selection or allocation bias. Also, several strategies were used to prevent patients and evaluator team from unblinding, formal assessment for awareness of the allocation (either active or placebo) was not performed. However, all outcomes assessed using different techniques (heat, pressure, sedation) were in the same direction and in dose-response gradient, hence unblinding is unlikely to have influenced the direction of our conclusions. ***iv***) New studies are needed to explore the pharmacokinetics parameters that could explain the subproportional increase of plasma melatonin according to exposure dose and the tendency to plateau. ***v***) Although a randomized clinical trial treating patients with endometriosis during two months using 10 mg at bed time did not report side effects that interfered with daily life activities [40], further randomized clinical trials would be required to assess better efficacy and possible side effects. Finally, although the dose-response effect was observed in these healthy subjects, further studies are required to test the dose-response effect of melatonin on clinical pain with diverse physiopathological mechanisms before any definitive conclusions can be drawn.

The present data indicate that sublingual melatonin exerts a well-defined dose-dependent antinociceptive activity, and there is a correlation between the plasma melatonin drug concentration and the acute changes in pain threshold. These results provide additional support for the investigation of melatonin as an analgesic agent.

## Supporting Information

Checklist S1 **CONSORT checklist.**
(DOC)

Protocol S1 **Trial protocol.**
(PDF)
