# Supplementary material for: A Phase II, Randomized, Double-Blind, Placebo Controlled, Dose-Response Trial of the Melatonin Effect on the Pain Threshold of Healthy Subjects
Source: PLoS One. 2013 Oct 2;8(10):e74107. doi: 10.1371/journal.pone.0074107 (PMC3788771; doi:10.1371/journal.pone.0074107)
Supplement: Checklist S1 — CONSORT checklist. (DOC) [file pone.0074107.s001.doc]

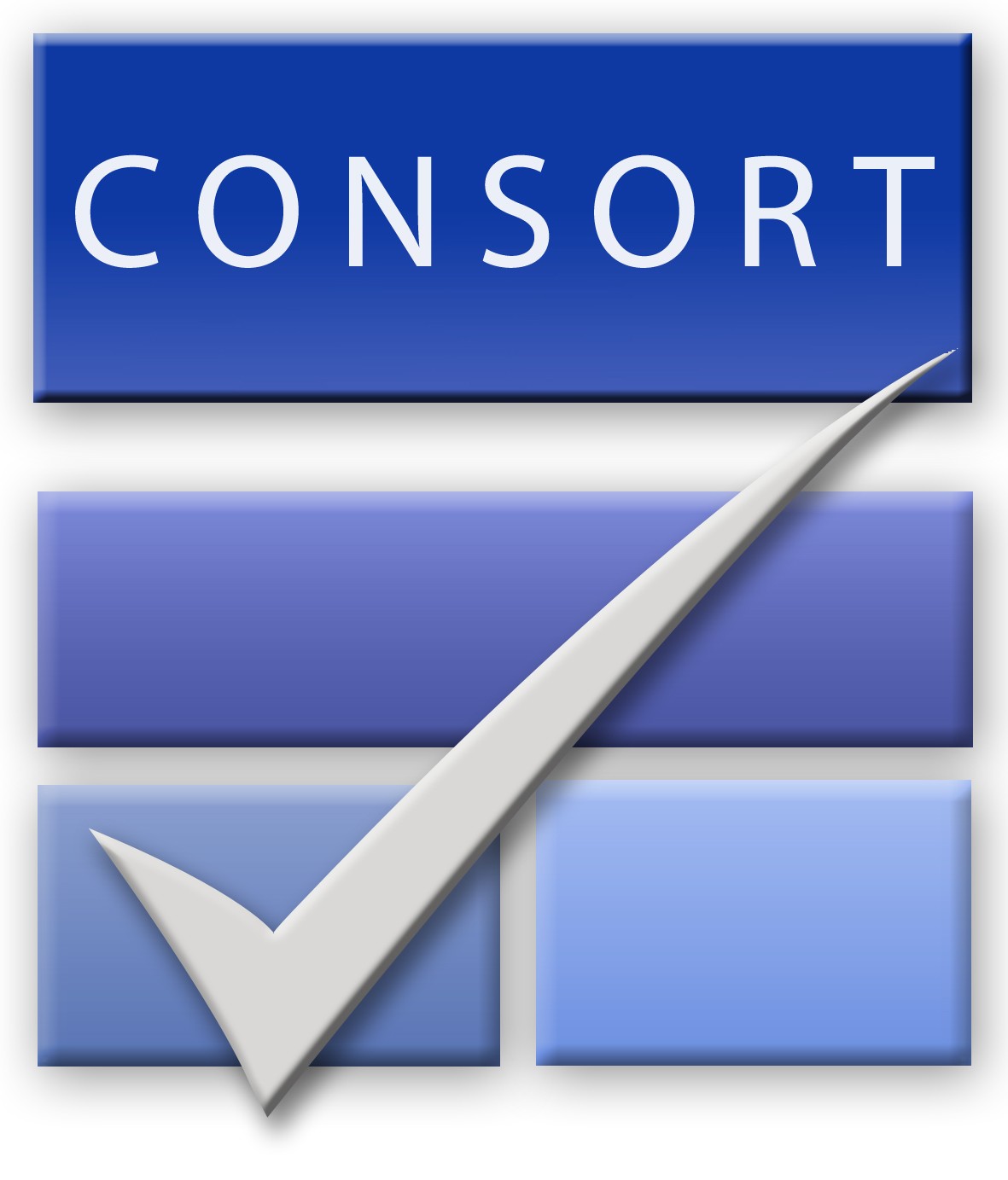
CONSORT 2010 checklist of information to include when reporting a randomised trial*

| Section/Topic | Item No | Checklist item | Reported on page No |
| --- | --- | --- | --- |
| Title and abstract | | | |
|  | 1a | **Identification as a randomised trial in the title** |  |
|  | 1b | **Structured summary of trial design, methods, results, and conclusions (for specific guidance see CONSORT for abstracts)** |  |
| Introduction | | | |
| Background and objectives | 2a | **Scientific background and explanation of rationale** |  |
|  | 2b | **Specific objectives or hypotheses** |  |
| Methods | | | |
| Trial design | 3a | **Description of trial design (such as parallel,** factorial) including allocation ratio |  |
|  | 3b | Important changes to methods after trial commencement (such as eligibility criteria), with reasons | N/A |
| Participants | 4a | **Eligibility criteria for participants** |  |
|  | 4b | **Settings and locations where the data were collected** |  |
| Interventions | 5 | **The interventions for each group with sufficient details to allow replication, including how and when they were actually administered** |  |
| Outcomes | 6a | **Completely defined pre-specified primary and secondary outcome measures, including how and when they were assessed** |  |
|  | 6b | Any changes to trial outcomes after the trial commenced, with reasons | N/A |
| Sample size | 7a | **How sample size was determined** |  |
|  | 7b | When applicable, explanation of any interim analyses and stopping guidelines | N/A |
| Randomisation: |  |  |  |
| Sequence generation | 8a | Method used to generate the random allocation sequence |  |
|  | 8b | Type of randomisation; details of any restriction (such as blocking and block size) |  |
| Allocation concealment mechanism | 9 | Mechanism used to implement the random allocation sequence (such as sequentially numbered containers), describing any steps taken to conceal the sequence until interventions were assigned |  |
| Implementation | 10 | Who generated the random allocation sequence, who enrolled participants, and who assigned participants to interventions |  |
| Blinding | 11a | **If done, who was blinded after assignment to interventions** (for example, participants, care providers, those **assessing outcomes**) **and how** |  |
|  | 11b | If relevant, description of the similarity of interventions |  |
| Statistical methods | 12a | **Statistical methods used to compare groups for primary and secondary outcomes** |  |
|  | 12b | Methods for additional analyses, such as subgroup analyses and adjusted analyses | N/A |
| Results | | | |
| Participant flow (a diagram is strongly recommended) | 13a | For each group, the numbers of participants who were randomly assigned, received intended treatment, and were analysed for the primary outcome |  |
|  | 13b | **For each group, losses and exclusions after randomisation, together with reasons** | **Figure 1** |
| Recruitment | 14a | Dates defining the periods of recruitment and follow-up | N/A |
|  | 14b | Why the trial ended or was stopped | N/A |
| Baseline data | 15 | **A table showing baseline demographic and clinical characteristics for each group** | **Table 1** |
| Numbers analysed | 16 | For each group, number of participants (denominator) included in each analysis and whether the analysis was by original assigned groups |  |
| Outcomes and estimation | 17a | **For each primary and secondary outcome, results for each group, and the estimated effect size and its precision (such as 95% confidence interval)** | **Table 2** |
|  | 17b | For binary outcomes, presentation of both absolute and relative effect sizes is recommended | N/A |
| Ancillary analyses | 18 | Results of any other analyses performed, including subgroup analyses and adjusted analyses, distinguishing pre-specified from exploratory | N/A |
| Harms | 19 | All important harms or unintended effects in each group (for specific guidance see CONSORT for harms) | N/A |
| Discussion | | | |
| Limitations | 20 | **Trial limitations, addressing sources of potential bias, imprecision, and, if relevant, multiplicity of analyses** |  |
| Generalisability | 21 | **Generalisability (external validity, applicability) of the trial findings** |  |
| Interpretation | 22 | **Interpretation consistent with results, balancing benefits and harms, and considering other relevant evidence** |  |
| Other information | | |  |
| Registration | 23 | Registration number and name of trial registry | U1111-1123-0192 |
| Protocol | 24 | Where the full trial protocol can be accessed, if available | **ReBec** |
| Funding | 25 | Sources of funding and other support (such as supply of drugs), role of funders | N/A |

[**www.consort-statement.org**](http://www.consort-statement.org/)**.**

*
