# Supplementary material for: A Phase II, Randomized, Double-Blind, Placebo Controlled, Dose-Response Trial of the Melatonin Effect on the Pain Threshold of Healthy Subjects
Source: PLoS One. 2013 Oct 2;8(10):e74107. doi: 10.1371/journal.pone.0074107 (PMC3788771; doi:10.1371/journal.pone.0074107)
Supplement: Protocol S1 — Trial protocol. (PDF) [file pone.0074107.s002.pdf]

UNIVERSIDADE FEDERAL DO RIO GRANDE DO SUL
FACULDADE DE MEDICINA

RESEARCH PROTOCOL - HOSPITAL DE CLÍNICAS DE PORTO ALEGRE

**Sponsor:**	Hospital de Clínicas de Porto Alegre
**Information provided by:**	Hospital de Clínicas de Porto Alegre
**Brazilian Clinical Trials Registry (ReBec): Identifier:**	U1111-1123-5109<br>http://www.ensaiosclinicos.gov.br/assistance/faq/
**Number of project in Institutional Review Board IRB 0000921:**	10-0921.

Keywords provided: melatonin; pain threshold; dose-response curve; clinical trial.

PURPOSE: To test the hypothesis that there are the melatonin dose response effect on pain threshold after taking into account the interindividual and intraindividual variability. We tested the pressure pain tolerance, the heat pain tolerance, and the sedative effect.

Condition	Intervention	Phase
Healthy subjects	Intervention groups:<br>1)sublingual melatonin 0.05mg/kg,<br>2)sublingual melatonin 0.15 mg /kg<br>3)sublingual melatonin 0.25 mg/kg<br>4) placebo	Phase II

Estimated Enrollment: 60 patients

**Study Type:**	Intervention
**Study Start Date:**	2011-05-01
**Completion Date:**	2011-11-01
**Study Design:**	Randomized, in parallel, double-blind, controlled with placebo
**Treatment duration:**	Two hours (One session)

**Arms**	**Assignment interventions**

Rua Ramiro Barcelos, 2400 – CEP 90035-003 – Porto Alegre/RS – Brasil
Fone: (51) 3359-1800. Fax: (51)3308.5232 – E-mail: hcpa.ufrgs.br.

Sublingual melatonin 0.05mg/kg		**Dose 0.05**
Sublingual melatonin 0.15 mg /kg		**Dose 0.15**
sublingual melatonin 0.25 mg/kg		**Dose 0.25**
Placebo		**Placebo**

Study allocation: Randomization in fixed block size of 12, stratified by gender

ENDPOINT CLASSIFICATION: Dose response-effect

INTERVENTION MODEL: Parallel Assignment

MASKING: Before the recruitment phase, the sealed envelopes containing the allocated treatment were prepared and numbered sequentially. The envelopes were only allowed to be opened after the subject signed the consent form; envelopes were opened by the nurse who administered the medications. Throughout the study period, randomization was performed by two investigators who were not involved in subject evaluation. Other individuals who were involved in patient care were unaware of the treatment group to which the patients belonged..

Primary purpose: Dose-response effect of melatonin in pain threshold

ELIGIBILITY Healthy

Ages Eligible for Study: 19 to 49 years

Genders Eligible for Study: Female and male

Accepts Healthy Volunteers Criteria Yes

STUDY ELIGIBILITY CRITERIA

Inclusion criteria

- Healthy subjects
- Age: 19 to 60 years

Exclusion criteria

Ccurrent acute or chronic pain ;
Use of analgesics in the past week;
Rheumatologic disease;
Clinically significant or unstable medical psychiatric disorder;
History of alcohol
Substance abuse in the past 6 months;
Neuropsychiatric comorbidity;
Use of central nervous system medications.

INVESTIGATORS

Principal Investigator: Wolnei Caumo MD, PhD.

Locations: Department: Laboratory of Pain & Neuromodulation

Rua Ramiro Barcelos, 2400 – CEP 90035-003 – Porto Alegre/RS – Brasil
Fone: (51) 3359-1800. Fax: (51)3308.5232 – E-mail: hcpa.ufrgs.br.

Institution: Hospital de Clínicas de Porto Alegre at UFRGS
Mailing address: Rua Ramiro Barcelos, 2350 - CEP 90035-003 Bairro Rio Branco - Porto Alegre – RS.
Phone: (55) 51- 3359.8083
Fax: (55) 51- 3359.8083
Email:caumo@cpovo.net

IRB: Research Ethics Committee at the Hospital de Clínicas de Porto Alegre.
Email:hcpa.ufrgs.br.
Phones: 55 (51) 3359-1800; 55 (51) 3359-8856.
Research Ethics Committee at the Hospital de Clínicas de Porto Alegre (Institutional Review Board IRB 0000921) (number: 10-0921).

THE PRIMARY OUTCOMES:

- Pain pressure threshold (PPT)
- Pain tolerance to PPT

THE SECONDARY OUTCOMES:

- Sedation level on VAS and BIS (bi-spectral index)
